# Minimally Invasive Coronary Artery Bypass Grafting in a Low-Risk Asian Cohort: A Propensity-Score Matched Study

**DOI:** 10.21470/1678-9741-2022-0421

**Published:** 2024-07-15

**Authors:** Zhi Xian Ong, Duoduo Wu, Jai Ajitchandra Sule, Guohao Chang, Faizus Sazzad, Haidong Luo, Peggy Hu, Theo Kofidis

**Affiliations:** 1 Department of Cardiac, Thoracic and Vascular Surgery, National University Heart Centre, Singapore; 2 Department of Surgery, Yong Loo Lin School of Medicine, National University of Singapore, Singapore

**Keywords:** Sternotomy, Coronary Artery Bypass, Propensity Matching, Atrial Fibrillation, Universities

## Abstract

**Introduction:**

Minimally invasive coronary artery bypass grafting (MICS CABG) offers a new paradigm in coronary revascularization. This study aims to compare the outcomes of MICS CABG with those of conventional median sternotomy CABG (MS CABG) within a growing minimally invasive cardiac surgical program in Singapore.

**Methods:**

Propensity matching produced 111 patient pairs who underwent MICS CABG or MS CABG between January 2009 and February 2020 at the National University Heart Centre, Singapore. Minimally invasive direct coronary artery bypass surgery patients were matched to single- or double-graft MS CABG patients (Group 1). Multivessel MICS CABG patients were matched to MS CABG patients with equal number of grafts (Group 2).

**Results:**

Overall, MICS CABG patients experienced shorter postoperative length of stay (*P*<0.071). In Group 2, procedural duration (*P*<0.001) was longer among MICS CABG patients, but it did not translate to adverse postoperative events. Postoperative outcomes, including 30-day mortality, reopening for bleeding, new onset atrial fibrillation as well as neurological, pulmonary, renal, and infectious complications were comparable between MICS and MS CABG groups.

**Conclusion:**

MICS CABG is a safe and effective approach for surgical revascularization of coronary artery disease and trends toward a reduction in hospital stay.

## INTRODUCTION

The conventional approach to coronary artery bypass grafting (CABG) via median sternotomy (MS) is invasive and often entails a prolonged recovery period lasting > 6 weeks to return to premorbid status. The current alternative of minimally invasive CABG (MICS CABG) has expanded from single vessel to multivessel coronary artery disease over the past decade^[1,2]^. MICS CABG presents a less invasive approach compared to MS CABG, yielding smaller incisions, reduced tissue trauma, and potentially expedited recovery periods for patients. Moreover, the smaller incisions characteristic of MICS CABG typically yield superior cosmetic results compared to the larger incisions necessitated by MS CABG, consequently enhancing patient satisfaction. Additionally, individuals undergoing MICS CABG may encounter shorter hospital stays and faster recovery times relative to counterparts undergoing conventional CABG, facilitating earlier resumption of daily activities^[3,4,5]^.

On the other hand, performing MICS CABG requires specialized skills due to the challenges of operating through smaller incisions. Surgeons must be proficient in advanced techniques, as MICS CABG has a steeper learning curve compared to traditional CABG. The limited visibility and maneuverability associated with MICS CABG may make complex procedures more challenging. Additionally, MICS CABG procedures may take longer and carry a risk of conversion to open surgery, which can increase complications and recovery time^[6,7,8,9]^.

Nevertheless, the advantages and disadvantages of MICS CABG have been limitedly compared to MS CABG in the past using propensity matching cohorts. Furthermore, there have been no evaluations of a multiracial Asian cohort to explicitly assess this comparison. The National University Heart Centre, Singapore, has established a comprehensive MICS CABG program, which includes multivessel coronary revascularization. This study aims to report early outcomes of MICS CABG and compare that to conventional MS CABG performed within a growing MICS CABG program at a centre with a moderate caseload.

## METHODS

One hundred and twelve patients underwent MICS CABG between January 2009 and June 2020 at the National University Heart Centre, Singapore. This study was approved by the local ethics review board (#2020/00547), and requirement for individual patient consent was waived. Propensity-score matching was carried out using a 0.1 caliper with 3,614 patients within the institution’s database who underwent conventional MS CABG between January 2009 and December 2018 (Table 1).

**Table 1:** Unmatched and matched groups.

Variables	Unmatched			Matched		
	MICS CABG (N=112)	MS CABG (N=3614)	Standard Mean Difference (%)	MICS CABG (N=111)	MS CABG (N=111)	Standard Mean Difference (%)
Age, years, mean	59.9	61.3	-15.2	59.8	59.4	< 10
Male (%)	88.4	83.2	-16.2	88.3	88.3	0
Diabetes (%)	41.1	54.	-27	41.4	47.8	-12.8
Cerebrovascular disease (%)	10.7	11.0	0.96	10.8	10.8	0
Peripheral vascular disease (%)	2.7	8.6	-36.9	2.7	0.9	-11.1
Ejection fraction category (%)						
Good (ò 50%)	77.7	58.7	45.6	78.4	79.3	< 10
Fair (30-49%)	17.9	31.0	-34.4	18.0	17.1	< 10
Poor (< 30%)	3.6	10.2	-35.9	3.6	3.6	0
Operative urgency (%)						
Elective	88.3	92.7	28.4	88.3	82.9	< 10
Urgent	11.7	7.3	-28.4	11.7	17.1	< 10
EuroSCORE II, mean (SD)	1.32	2.95	56.4	1.31	1.28	< 10

CABG=coronary artery bypass grafting; EuroSCORE=European System for Cardiac Operative Risk Evaluation; MICS CABG=minimally invasive CABG; MS=median sternotomy; SD=standard deviation

Minimally invasive direct coronary artery bypass (MIDCAB) patients were matched to single/double vessel MS CABG patients (Group 1) due to scarcity of single vessel MS CABG performed. Multivessel MICS CABG patients were propensity matched graft-for-graft to MS CABG patients (Group 2). Baseline characteristics, intraoperative data, and 30-day postoperative outcomes were compared between MICS and MS CABG groups.

### Primary and Secondary Outcomes

The primary outcome of this study was postoperative length of stay. Secondary outcomes included operative times, 30-day mortality, and postoperative complications including reopening for bleeding, new onset atrial fibrillation, and neurological, renal, pulmonary, and infectious complications. Stroke was defined as a permanent neurological deficit associated with an ischaemic infarct or intracranial haemorrhage on radiological imaging. Prolonged ventilation was defined as requiring > 24 hours of ventilation. Renal impairment was defined as a rise in creatinine above the upper limit of baseline. Surgical site infection was defined as sternal infections for MS CABG and thoracotomy/cannulation site infections for MICS CABG. Non-surgical infections comprised urinary tract infection or septicaemia.

### Statistical Analysis

All statistical analyses were performed using R Studio (RStudio Team 2015, Boston, Massachusetts, United States of America) software. Categorical data were represented as frequencies and percentages. Continuous data were tested for normality via Shapiro-Wilk’s method. Normally distributed continuous variables were expressed as mean (standard deviation). Propensity scores between the MICS CABG and database patients were estimated using logistic regression with 1:1 matching. MS CABG patients with poor matching propensity scores were excluded from the analysis. For non-matched cohorts, categorical variables were compared using the Chi-square test while continuous variables were analysed using the Student’s *t*-test or Mann-Whitney U test where appropriate. For propensity-score matched pairs, categorical variables were compared using McNemar’s test, and continuous variables were compared using Wilcoxon’s paired signed-rank test.

### Surgical Technique

Most patients in the MS group underwent conventional on-pump CABG with individual aorto-coronary anastomosis performed via side-clamping of the aorta. Few patients in the MS group underwent off-pump or on-pump beating CABG. MICS CABG patients underwent either MIDCAB or multivessel grafting via left anterior mini-thoracotomy. The left internal mammary artery (LIMA) was taken down *in situ* in a pedicled fashion under direct vision through left anterior mini-thoracotomy using a combination of electrocautery and ultrasonic dissection (Harmonic Synergy®). The Rultract® retractor system (Rultract, Ohio, United States of America) coupled with the Thoratrak™ MICS CABG retractor (Medtronic, Minnesota, United States of America) was used for intercostal retraction and elevation of the left hemithorax to provide adequate exposure. MIDCAB was indicated in patients who had single-vessel left anterior descending artery (LAD) stenosis.

MIDCAB surgeries were predominantly performed off-pump, grafting the LIMA to the LAD with the LAD target stabilised using an Octopus™ Nuvo or Octopus™ Evolution stabiliser (Medtronic, Minnesota, United States of America). Multivessel MICS CABG surgeries were per formed either on an arrested heart or on a beating heart with peripheral cardiopulmonary bypass (CPB) support. If the heart was arrested, a Chitwood® cross-clamp (Scanlan International, Inc, Minnesota, United States of America) was inserted via a left axillary stab incision, and antegrade cardioplegia was administered using a Miar™ cannula (Medtronic, Minnesota, United States of America). For multivessel MICS CABG performed on a beating heart, coronary targets were stabilised using an Octopus™ Nuvo stabiliser with or without a Starfish™ heart positioner (Medtronic, Minnesota, United States of America).

CPB was performed using standard aortic and two-stage right atrial cannulation for MS CABG cases, while femoral arterial and venous cannulations were used in on-pump MICS CABG cases. In all multivessel CABG cases, CABG was performed first with the right coronary artery target, followed by obtuse marginal, ramus, or diagonal, where applicable, and lastly, the LAD. LIMA was the default conduit to graft the LAD, while saphenous vein or left radial artery grafts were used for the remaining targets. All distal anastomoses were performed conventionally under direct vision with continuous 7-0 polypropylene sutures.

## RESULTS

There were 111 propensity-matched pairs. Baseline characteristics are summarised in Supplementary Table 1. Patient demographics within the propensity-matched groups were comparable. Institutional caseload for MICS CABG and CPB times are demonstrated in Figures 1A and 1B. Distribution of the grafts in MICS CABG are also shown in Figure 2.

**Supplementary Table 1:** Patient demographics.

Variables	Overall			Single/Double Graft(s) (Group 1)			Multivessel (Group 2)		
	MICS CABG (N=111)	MS CABG (N=111)	*P*-value	MIDCAB^a^ (N=64)	MS CABG (N=64)	*P*-value	MICS CABG (N=46)	MS CABG (N=46)	*P*-value
Age, years, mean (SD)	59.8 (9.7)	59.4 (7.7)	0.737	58.3 (9.8)	57.7 (7.9)	0.737	60.9 (9.4)	61 (8.0)	0.953
Male (%)	98 (88.3)	98 (88.3)	1.00	55 (85.9)	54 (84.4)	1.00	43 (93.5)	43 (93.5)	1.00
Race (%)			0.990			0.540			0.435
Chinese	76 (67.6)	76 (68.5)		41 (64.1)	37 (57.8)		34 (73.9)	27 (58.7)	
Indian	13 (11.7)	13 (11.7)		8 (12.5)	12 (18.8)		4 (8.7)	6 (13)	
Malay	18 (16.2)	18 (16.2)		12 (18.8)	14 (21.9)		7 (15.2)	10 (21.7)	
Others	4 (3.6)	5 (4.5)		3 (4.7)	1 (1.6)		1 (2.2)	3 (6.5)	
Smokers (%)	26 (23.4)	26 (23.4)	1.00	16 (25)	19 (30.2)	0.556	26 (56.5)	25 (54.3)	1.00
Diabetes (%)	46 (41.1)	53 (47.7)	0.418	27 (42.2)	25 (39.1)	0.857	19 (41.3)	13 (28.3)	0.274
Hypertension (%)	82 (73.9)	89 (80.2)	0.338	47 (73.4)	50 (78.1)	0.680	36 (78.3)	41 (89.1)	0.259
Hyperlipidaemia (%)	91 (82)	103 (92.8)	0.025	55 (85.9)	55 (85.9)	1.00	33 (71.7)	38 (82.6)	0.321
Renal disease (%)	9 (8.1)	3 (2.7)	0.135	8 (12.5)	2 (3.1)	0.096	1 (2.2)	3 (6.5)	0.617
COPD (%)	3 (2.7)	1 (0.9)	0.175	1 (1.6)	0	0.603	0	1 (2.2)	1.00
Cerebrovascular disease (%)	12 (10.8)	12 (10.8)	1.00	8 (12.5)	9 (14.1)	1.00	2 (4.3)	1 (2.2)	1.00
Previous PCI (%)	37 (33.3)	23 (20.7)	0.049	20 (31.1)	16 (25)	0.556	17 (37)	11 (23.9)	0.257
Peripheral vascular disease (%)	3 (2.7)	1 (0.9)	0.622	1 (1.6%)	0	1.00	1 (2.2)	1 (2.2)	1.00
Ejection fraction, mean (SD)	54.9 (11)	55.3 (12)	0.778	55.4 (11.9)	53.2 (12.6)	0.303	53.7 (10.3)	53.7 (10.9)	0.998
Ejection fraction category (%)			0.984			0.893			0.550
Good (ò 50%)	87 (78.4)	88 (79.3)		50 (78.1)	48 (75)		35 (76.1)	34 (73.9)	
Fair (30-49%)	20 (18)	19 (17.1)		11 (17.2)	12 (18.8)		10 (21.7)	12 (26.1)	
Poor (< 30%)	4 (3.6)	4 (3.6)		3 (4.7)	4 (6.3)		1 (2.2)	0	
Preoperative IABP (%)	0	3 (2.7)	0.247	0	4 (6.3)	0.119	0	4 (8.7)	0.117
Preoperative NYHA II and above (%)	52 (46.8)	43 (38.7)	0.278	29 (45.3)	21 (32.8)	0.205	22 (47.8)	18 (39.1)	0.528
EuroSCORE II, mean (SD)	1.31 (1.28)	1.28 (1.16)	0.850	1.31 (1.49)	1.38 (1.43)	0.786	1.29 (0.92)	1.37 (0.95)	0.687

CABG=coronary artery bypass grafting; COPD=chronic obstructive pulmonary disease; EuroSCORE=European System for Cardiac Operative Risk Evaluation; IABP=intra-aortic balloon pump; MICS CABG=minimally invasive CABG; MIDCAB=minimally invasive direct coronary artery bypass; MS=median sternotomy; NYHA=New York Heart Association; PCI=percutaneous coronary intervention; SD=standard deviation

a MIDCAB patients were matched to MS patients with single or double vessel CABG with via propensity score matching

**Fig. 1A:**
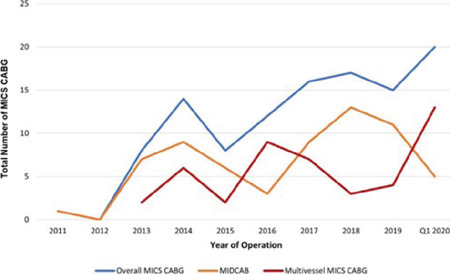
Minimally invasive coronary artery bypass grafting (MICS CABG) over years. MIDCAB=minimally invasive direct coronary artery bypass.

**Fig. 1B:**
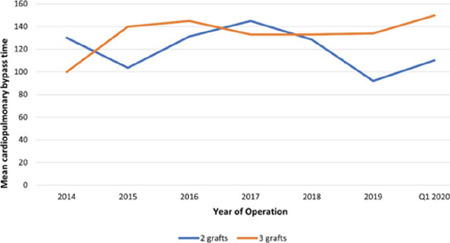
Mean cardiopulmonary bypass time over years in Group 2.

**Fig. 2:**
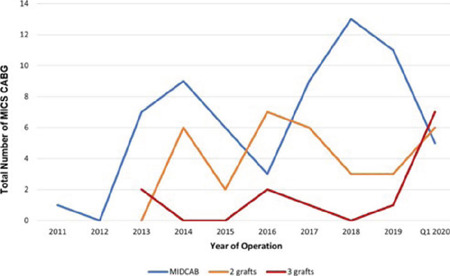
Distribution of cases over years by the number of grafts. MICS CABG=minimally invasive coronary artery bypass grafting; MIDCAB=minimally invasive direct coronary artery bypass.

### Intraoperative Details

Procedural details of matched pairs are shown in Table 2. Most MIDCAB patients underwent off-pump and on-pump beating heart surgery (*P*<0.001). In Group 2, on-pump beating procedures were more common among MICS CABG patients (*P*<0.001). CPB time (*P*=0.005) and procedure durations (*P*<0.001) were significantly longer in MICS CABG patients.

**Table 2:** Procedure and intraoperative data.

Variables	Overall			Non-multivessel (Group 1)			Multivessel (Group 2)		
	MICS CABG (N=111)	MS CABG (N=111)	*P*-value	MIDCAB^a^ (N=64)	MS CABG (single/ double) (N=64)	*P*-value	MICS CABG (N=46)	MS CABG (N=46)	*P*-value
Operative urgency (%)			0.339			1.00			1.00
Elective	98 (88.3)	92 (82.9)		64 (100)	64 (100)		39 (84.8)	39 (84.8)	
Urgent	13 (11.7)	19 (17.1)		0	0		7 (15.2)	7 (15.2)	
CABG category (%)			< 0.001			< 0.001			< 0.001
Off-pump	56 (50.5)	2 (1.8)		54 (84.4)	7 (10.9)		1 (2.2)	3 (6.5)	
On-pump beating	33 (29.7)	2 (1.8)		8 (12.5)	3 (4.7)		26 (56.5)	1 (2.2)	
Cardioplegic arrest	22 (19.8)	107 (96.4)		2 (3.1)	54 (84.4)		19 (41.3)	42 (91.3)	
Cardiopulmonary bypass duration, mean (SD)	131.2 (42.7)	136.3 (46.1)	0.62	-	-	-	145.5 (70.6)	104.3 (48.4)	0.005
Aortic cross-clamping duration, mean (SD)	64.7 (13.1)	80.5 (23.7)	0.037	-	-	-	59.1 (15.7)	52.9 (20)	0.434
Length of procedure, mean (SD)	286.3 (85.1)	272.8 (69.6)	0.24	234.3 (57.5)	239 (49.2)	0.62	359.7 (63.1)	233.5 (36.3)	< 0.001

CABG=coronary artery bypass grafting; MICS CABG=minimally invasive CABG; MIDCAB=minimally invasive direct coronary artery bypass; MS=median sternotomy; SD=standard deviation

a MIDCAB patients were matched to MS patients with single or double vessel CABG with propensity score matching.

### Postoperative Outcomes

Thirty-day mortality rates and perioperative complications were comparable between MICS and MS CABG patients (Table 3). Overall, postoperative length of stay was generally shorter amongst MICS CABG patients. Rates of reoperation and neurological complications were generally low in all patients.

**Table 3:** Postoperative outcomes of matched pairs.

Variables	Overall			Non-multivessel (Group 1)			Multivessel (Group 2)		
	MICS CABG (N=111)	MS CABG (N=111)	*P*-value	MIDCAB^a^ (N=64)	MS CABG (N=64)	*P*-value	MICS CABG (N=46)	MS CABG (N=46)	*P*-value
Postoperative length of stay, median (IQR)	6 (2)	7 (3)	< 0.001	5 (2)	7 (5)	< 0.001	6 (5.7)	7 (2.25)	0.288
Conversion to median sternotomy (%)	7 (6.3)	-	-	2 (3.1)	-	-	5 (10.9)	-	-
Reopening (%)	5 (4.5)	4 (3.6)	1.00	2 (3.1)	4 (6.3)	0.687	3 (6.8)	2 (4.7)	1.00
Permanent pacemaker (%)	0	0	-	0	1 (1.6)	-	0	1 (2.2)	-
New-onset atrial fibrillation (%)	12 (10.8)	15 (13.5)	0.701	5 (7.8)	8 (12.5)	0.549	7 (15.2)	6 (13)	1.00
Postoperative IABP (%)	2 (1.8)	0	-	1 (1.6)	0	-	1 (2.2)	0	-
Neurological complications^b^ (%)	1 (0.9)	4 (3.6)	0.375	0	2 (3.1)	-	1 (2.2)	2 (4.7)	1.00
Surgical site infections (%)	2 (1.8)	4 (3.6)	0.687	1 (1.6)	1 (1.6)	1.00	1 (2.2%)	0	-
Non-surgical site infections^c^ (%)	3 (2.7)	2 (1.8)	1.00	1 (1.6)	2 (3.1)	1.00	1 (2.2)	1 (2.2)	1.00
Prolonged ventilation^d^ (%)	3 (2.7)	3 (2.7)	1.00	0	3 (4.7)	-	3 (6.8)	1 (2.2)	0.625
Pneumonia (%)	4 (3.6)	2 (1.8)	0.687	1 (1.6)	3 (4.7)	0.50	3 (6.8)	1 (2.2)	0.625
Pleural effusion requiring drainage (%)	0	0	-	0	1 (1.6)	-	0	0	-
Acute renal injury (%)	2 (1.8)	2 (1.8)	1.00	1 (1.6)	5 (7.8)	0.125	1 (2.2)	1 (2.2)	1.00
30-day mortality (%)	0	0	1.00	0	1 (1.6)	-	0	1 (2.2)	-

CABG=coronary artery bypass grafting; IABP=intra-aortic balloon pump; IQR=interquartile range; MICS CABG=minimally invasive CABG; MIDCAB=minimally invasive direct coronary artery bypass; MS=median sternotomy

^a^MIDCAB patients were matched to MS patients with single or double vessel CABG with propensity score matching; ^b^Comprises permanent stroke, transient ischemic attack, delirium;^c^Urinary tract infection or sepsis; ^d^Prolonged ventilation defined as ventilation > 24 hours postoperatively

## DISCUSSION

In our MICS CABG program, we report comparable perioperative outcomes in MICS CABG patients with a shorter postoperative length of stay. The longer procedural times for MICS CABG are consistent with other studies^[10,11]^. This is attributed to technical challenges associated with a much smaller access and a learning curve for MICS CABG. This observation of longer operative time did not translate to any clinical significance.

Previous studies showed that MICS CABG is associated with less postoperative complications, such as new onset atrial fibrillation and surgical site infections^[12,13,14,15,16]^. The Sternotomy Versus Thoracotomy (or STET) trial reported rates of postoperative arrhythmias and not just atrial fibrillation. It showed a higher incidence of arrhythmia among MS off-pump CABG patients than thoracotomy CABG patients^[5,11]^. This was comparable in this stringent propensity-matched study.

Single lung ventilation in the setting of MICS CABG did not increase risk of pulmonary complications. This is consistent with a previous review of five non-randomised control trials which demonstrated that postoperative lung function in patients with known respiratory problems is better with MICS CABG^[17]^. More recently, continuous full-lung ventilation during MICS CABG which improves postoperative lung function has been described^[9,10]^. More studies are warranted to determine its efficacy.

The shorter postoperative length of stay among MICS CABG patients was consistently reported in the literature^[10,11]^. This can be attributed to the shorter recovery needed with smaller incisions. Reduced surgical trauma and strict postoperative protocols in physiotherapy in our institution could be contributing factors. Despite this, it is important to note that discharge protocols from intensive care unit and from the hospital vary between centres.

Whilst conferring the benefits of MICS CABG, the reduced utility of conventional on-pump techniques may yield additional benefits associated with reduced systemic inflammatory response and reduced manipulation of the aorta^[18,19]^. The Randomized On/Off Bypass (or ROOBY) and CABG Off or On Pump Revascularization Study (or CORONARY) trials demonstrated similar outcomes between CABG performed off-pump *versus* on-pump^[18,20]^. In our institution, off-pump procedures are mainly reserved for MS CABG in patients who have a hostile aorta due to institutional practice.

### Limitations

Firstly, the sample size was not powered for non-inferiority. This was mitigated by the stringent criteria of propensity matching. Secondly, this was a retrospective study with some missing data for patients operated prior to 2015.

## CONCLUSION

This study demonstrates that MICS CABG is a safe and effective alternative to conventional MS CABG and is likely to enhance recovery. More prospective follow-up data is required to validate the findings of this study. Our moderate but increasing case volume may provide a better perspective on our performance in future studies.

## Abbreviations, Acronyms & Symbols

CABG: Coronary artery bypass grafting
COPD: Chronic obstructive pulmonary disease
CPB: Cardiopulmonary bypass
EuroSCORE: European System for Cardiac Operative Risk Evaluation
IABP: Intra-aortic balloon pump
IQR: Interquartile range
LAD: Left anterior descending artery
LIMA: Left internal mammary artery
CABG: Coronary artery bypass grafting
MICS: Minimally invasive cardiac surgery
MICS CABG: Minimally- invasive coronary artery bypass grafting
MIDCAB: Minimally invasive direct coronary artery bypass
MS: Median sternotomy
NYHA: New York Heart Association
PCI: Percutaneous coronary intervention
SD: Standard deviation
